# Impact of the South Atlantic Anomaly on radiation exposure at flight altitudes during solar minimum

**DOI:** 10.1038/s41598-023-36190-5

**Published:** 2023-06-08

**Authors:** Matthias M. Meier, Thomas Berger, Thomas Jahn, Daniel Matthiä, Mona C. Plettenberg, Markus Scheibinger, Kai Schennetten, Michael Wirtz

**Affiliations:** 1 German Aerospace Center, Institute of Aerospace Medicine, Radiation Biology, Cologne, Germany; 2 Lufthansa German Airlines, Lufthansa Basis, Frankfurt/Main, Germany

**Keywords:** Applied physics, Environmental impact

## Abstract

The South Atlantic Anomaly (SAA) is a geographical region over the South Atlantic Ocean where the inner Van Allen radiation belt extends down particularly close to Earth. This leads to highly increased levels of ionizing radiation and related impacts on spacecraft in Low Earth Orbits, e.g., correspondingly increased radiation exposure of astronauts and electronic components on the International Space Station. According to an urban legend, the SAA is also supposed to affect the radiation field in the atmosphere even down to the altitudes of civil aviation. In order to identify and quantify any additional contributions to the omnipresent radiation exposure due to the Galactic Cosmic Radiation at flight altitudes, comprehensive measurements were performed crossing the geographical region of the SAA at an altitude of 13 km in a unique flight mission—Atlantic Kiss. No indication of increased radiation exposure was found.

## Introduction

**Figure 1:**
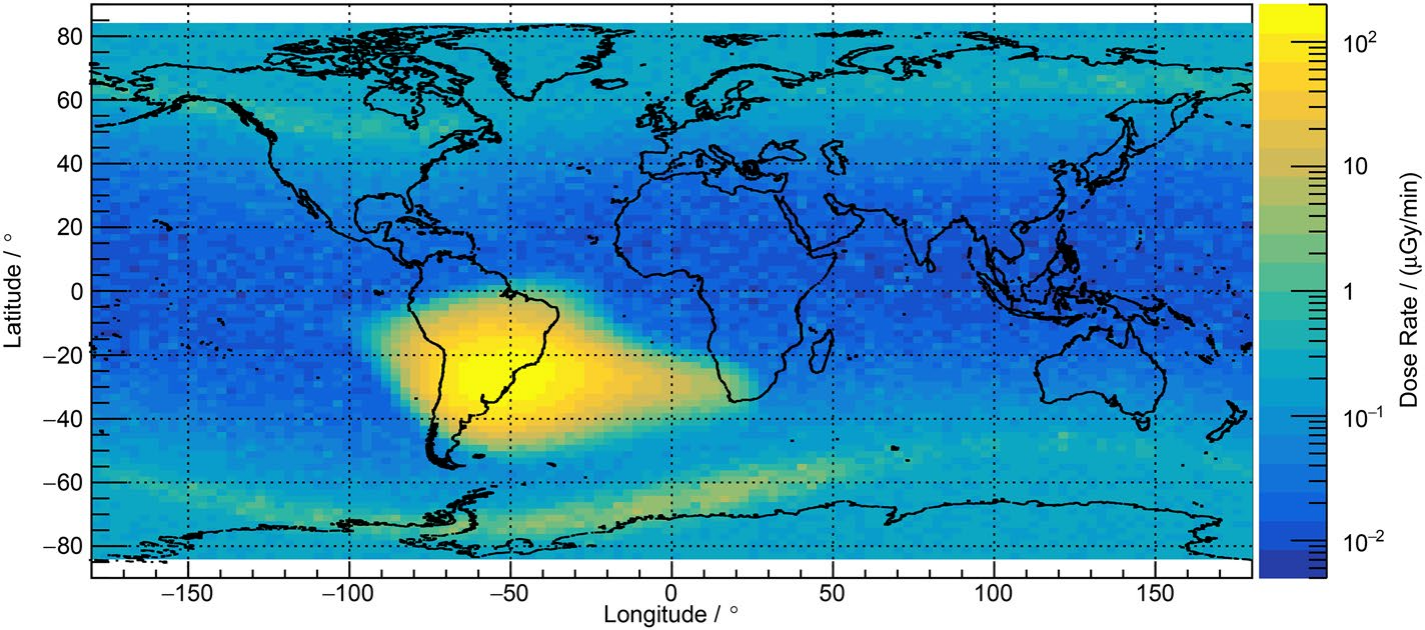
Absorbed dose rate (µGy/min) in silicon at 600 km altitude measured with the RAMIS instrument aboard the DLR Eu:CROPIS satellite in March and April 2021.

The Earth is effectively shielded from cosmic radiation by its magnetic field and its atmosphere^1^. The geomagnetic field resembles a dipole field at some distance from the Earth’s surface. Charged particles from outer space and the decay products of neutrons generated in the atmosphere are trapped in the so-called Van Allen radiation belts surrounding the Earth. The axis of the Earth’s dipole shaped magnetic field, however, is shifted and tilted with respect to the Earth’s rotational axis, which results in the phenomenon of the so-called South Atlantic Anomaly (SAA). It spans the South Atlantic Ocean from Africa to South America and from the Equator to Antarctica and can be characterized as a region with decreased geomagnetic field strength relative to comparable latitudes^2^. As a result, the inner Van Allen radiation belt extends down particularly close to Earth, which leads to highly increased radiation levels in near-Earth space in the region. This effect poses a safety hazard to human space flight and to electronic components, e.g., on the ISS. Increased levels of ionizing radiation during SAA transits had already been measured aboard the Russian Space Station MИP (“peace”)^3^ and aboard the ISS^4–6^. Inside the ISS, which is circling the Earth at an altitude of about 420 km, the average daily dose rates in silicon measured in the March/April 2021 timeframe accounted to some 280 µGy/day with peak values reaching several hundred µGy/min close to the center of the SAA. Dose values even increase at higher altitudes and lower geomagnetic shielding, e.g., observed with the RAMIS (RAdiation Measurements In Space) detector aboard the DLR Eu:CROPIS (Euglena gracilis: Combined Regenerative Organic-food Production In Space) satellite in a polar orbit at around 600 km^7^. Figure 1 shows the map of the dose rates in silicon for the March/April 2021 timeframe behind an average shielding of 3 mm aluminium measured by RAMIS up to a latitude of ± 83°. The geographical region of the SAA is characterized by the strong increase in the dose rates in silicon with peak values up to 180 µGy/min within the core of the SAA.

Several examples of radiation damage on satellites passing through the SAA are given by Olson and Amit, who also raise the question if there are health issues posed by the SAA even at lower altitudes, i.e., “in the 5–10 km altitude range of commercial jet travel”^2^. In an article of the German popular scientific journal “Bild der Wissenschaft”, the rumor was planted that the inner Van Allen radiation belt would affect the radiation exposure at the altitudes of civil aviation in the South Atlantic region as well and that the radiation dose on a flight from Frankfurt to Buenos Aires would be about 1000 times as much as on a flight to Tokyo, albeit no scientific evidence for this statement is given in that article^8^. Nevertheless, this rumor quickly spread among German aircrew members and has been a cause of concern since then. Although measuring values at aviation altitudes from the South Atlantic region published by Federico et al. in 2015 gave no indication of locally increased dose rates^9^, these rumours were further fostered, e.g., by an article available on the webpage of the German Research Centre for Geosciences (GFZ) suggesting that “… protection from harmful radiation from space is reduced” in the SAA region which leads to “… higher doses of radiation for passengers of long-distance flights”^10^. Moreover, even conspiracists have claimed that the public would be intentionally misinformed about the purported highly increased levels of radiation exposure at an altitude of 12 km in the geographical region of the SAA^11^. Further alarmist or misleading information, i.e., not excluding aviation and lower altitudes when referring to effects in Low Earth Orbit (LEO), is spread on the internet, e.g., by Wikipedia using the search term “SAA”. For instance, the statement „Auch auf der Erdoberfläche ist die ionisierende Strahlung erhöht“ (“the ionizing radiation is even increased at ground level“, Wikipedia German version, accessed on 27 February 2023)^12^ in the context with the SAA is misleading, since the measurable increase in ionizing radiation at ground level is caused by terrestrial radioactivity, e.g., ^232^Th^13,14^. However, this phenomenon of misleading information is not restricted to the German speaking countries, for example “Pour une altitude donnée, le niveau de radiations en provenance de l'espace est plus élevé dans l'Atlantique sud qu'en d'autres points du globe” (“At a given altitude, the level of radiation coming from space is higher in the South Atlantic than in other points of the globe”, Wikipedia French version, accessed on 27 February 2023)^15^ or “Omdat het aardmagneetveld zwakker wordt met zo'n 5% per eeuw, neemt de bescherming door de Van Allen-gordels ook af en komt de ZAA steeds lager boven het aardoppervlak te liggen” (“Since the geomagnetic field is weakening by some 5% per century, the shielding of the Van Allen belts is likewise decreasing and the SAA is reaching closer and closer to the Earth’s surface”, Wikipedia Dutch version, accessed on 27 February 2023)^16^. Although the urban legend of higher doses of radiation at flight altitudes in the geographical region of the SAA might cause concern among passengers and aircrew, it has not been supported by any scientific evidence yet.

Radiation exposure of aircrew and corresponding radiation protection measures have been legally regulated in several countries with superior occupational safety standards based on the recommendations by the International Commission on Radiological Protection (ICRP) for many years^17–20^. The regulations include, among other things, monitoring of occupational radiation exposures and compliance with stipulated dose limits. In practice, dose assessment is carried out using atmospheric radiation models that have to take all relevant components contributing to the radiation field at aviation altitudes into account. Therefore, a potentially substantial component caused by the inner Van Allen radiation belt in the geographical region of the SAA needs to be quantified and included in such models, if required. The German Aerospace Center (DLR) performs operational dose calculations for several airlines, e.g., Lufthansa German Airlines, using the PANDOCA (Professional AviatioN DOse CAlculator) model that has been successfully checked with measuring values acquired during regular measuring flights all over the world for quality assurance. However, in the geographical region of the SAA it had only been verified up to an altitude of 10,500 m so far^21^. Since PANDOCA does not include any potential radiation effects due to the SAA and related dose contributions, the acquisition of measurements at higher altitudes and comparison with PANDOCA model calculations is a practical method to identify any additional radiation component in this region. In order to answer the question whether the radiation field of the SAA touches the upper airspace of civil aviation or not, i.e., if it causes an increase in radiation exposure in comparison with the omnipresent galactic radiation component as assessed with PANDOCA in the geographical region of interest, the mission Atlantic Kiss was planned as a comprehensive measuring flight from Frankfurt (FRA) to Buenos Aires (EZE) for June 2020 close to the transition between solar cycle 24 and 25, i.e., during solar minimum conditions. However, this realization of the Atlantic Kiss mission had to be cancelled due to the worldwide SARS-CoV-2 (Severe Acute Respiratory Syndrome CoronaVirus 2) pandemic crisis that set in shortly after the planning had been finished.

## Atlantic Kiss—Mission (im-)possible

The worldwide SARS-CoV-2 crisis did not only affect aviation related research but also posed a challenge to the Alfred Wegener Institute (AWI), the German Centre for Polar and Marine Research, since the scientists and crew of the research vessel Polarstern and the Antarctic research station Neumayer III had to be exchanged in a comparatively safe place near Antarctica at the end of the Antarctic summer in 2021. The Falkland Islands appeared to be one of the limited options to this end and the management of Lufthansa German Airlines (DLH) was already contacted in July 2020 by AWI with the request to examine if a nonstop flight from Hamburg (HAM) to the Falkland Islands would be possible. A detailed analysis of the situation showed that a flight from HAM to Mount Pleasant (MPN, East Falkland) using an Airbus A350 and a respective return flight from MPN to Munich (MUC) was deemed feasible. This also offered a unique opportunity to involve DLR and finally make the realization of the flight mission Atlantic Kiss through the geographical region of the SAA possible.

### Dose quantities

The complex radiation field at aviation altitudes is generated by impinging primary particles of cosmic origin and their interactions with the atmosphere and consists of various radiation components such as protons, neutrons, electrons, pions, muons, etc. The effects of radiation are characterized by the deposition of its energy in matter and different dose quantities have been defined for this purpose. The fundamental quantity is the absorbed dose given by the absorbed energy per mass. Since the underlying interactions depend on the absorbing material, an identical exposure situation in a radiation field leads to different values for the absorbed dose in different materials. In order to investigate potential effects of the SAA on the radiation exposure at aviation altitudes, three measurable dose quantities were compared with the corresponding predictions of PANDOCA model calculations, namely the ambient dose equivalent H*(10), the neutron component H_N_ of the ambient dose equivalent H*(10), and the absorbed dose in silicon D_Si_. The ambient dose equivalent H*(10) is an operational quantity based on the absorbed dose in tissue and a radiation quality factor depending on the ionization density of the radiation field^20^. H*(10) is a conservative estimator for the effective dose at flight altitudes, i.e., the primary protection quantity in occupational radiation protection of aircrew^22^. The neutron component H_N_ is of particular interest due to its important contribution to H*(10). Furthermore, neutrons can have greater ranges in the atmosphere than the primary charged cosmic particles that generate them. Therefore, neutrons are messengers of particle interactions in the upper atmosphere and can even reach the ground where they are detected by Neutron Monitors (NM), which provide general information on the radiation intensity in the atmosphere^23^. Further valuable information can be deduced from the absorbed dose in silicon D_Si_. Silicon detectors are particularly susceptible to charged particles and not very sensitive to neutrons^24^. Thus, D_Si_ complements H_N_ with information about the non-neutron component of the radiation field.

An impact of the SAA on the radiation field at flight altitudes would be assumed if a significant deviation of the measuring values from PANDOCA model calculations^21^ were observed for any dose quantity taking the respective uncertainties into account. The PANDOCA model provides calculated dose rates at aviation altitudes considering solar modulation, geomagnetic shielding, and atmospheric shielding. Uncertainties in the model calculations arise from uncertainties in the primary GCR spectra, the transport calculations of the primary and secondary cosmic radiation in the atmosphere, and the effective vertical cut-off rigidities for the magnetic shielding. The combined uncertainties of the model for the calculation of the exposures from GCR have been estimated by numerous measuring campaigns^21,28,32,35^ to be about 5% to 10%. The comparison of the measurements of the different dose quantities with the corresponding model predictions, which reflect our current knowledge of the radiation exposure due to GCR, can help identify additional contributions to the radiation field.

### Measuring instruments

The instruments used to measure the three different dose quantities H*(10), H_N_, and D_Si_ of the radiation field at aviation altitudes during the mission Atlantic Kiss were two types of Tissue Equivalent Proportional Counters (TEPC), a Berthold Neutron Probe LB6411-Pb with extended energy range as well as two Liulin-6G silicon semiconductor detectors.

#### HAWK

The two Tissue Equivalent Proportional Counters deployed were HAWK environmental radiation monitors version 2 and 3 manufactured by Far West Technology Inc. These instruments measure time resolved spectra of lineal energy which allow the determination of the ambient dose equivalent H*(10) and its corresponding dose rate. The sensitive volume of these detectors consists of a sphere with a diameter of 127 mm made of tissue equivalent plastic (A-150) and filled with propane gas at low pressure. It is designed to mimic the energy deposition in 2 µm tissue. The signals of the detected particles are processed using two multi-channel-analyzers (MCA) with two different gains to measure spectra of the lineal energy transfer. For the calculation of the dose equivalent, it is assumed that these spectra can be directly compared to the corresponding linear energy transfer (LET) spectra at aviation altitudes^4^. The low-gain analogue to digital converter (ADC) measures LET spectra up to 1535 keV/μm with a resolution of 1.5 keV/µm per channel and the high-gain ADC up to 25.6 keV/µm with 0.1 keV/µm per channel. Both instruments were calibrated at the Pacific Northwest National Laboratory by the manufacturer with sources that are traceable to the standards by the National Institute of Standards and Technology (NIST). The calibration was checked with an external Cs-137 source^25,26^.

#### Neutron probe LB6411-Pb

The neutron probe LB6411-Pb was designed by Berthold Technologies to measure the neutron component H_N_ of the ambient dose equivalent H*(10) and the corresponding dose rate. It consists of a sphere of polyethylene with a diameter of 25 cm to thermalize neutrons which are detected by a Helium-3 proportional counter tube. The detector has an outer layer of 10 mm lead to enhance the response to high energy neutrons by creating secondary neutrons with lower energies due to spallation processes. The instrument was calibrated at the CERN-EU high-energy Reference Field (CERF) facility by the manufacturer^27^. The rate of the ambient dose equivalent is recorded in intervals of five minutes and displayed. These data are of special interest since they allow the assessment of the neutron component of the radiation field at aviation altitudes and any unexpected effects already during the flight.

#### Liulin-6G

Two semiconductor detectors of type Liulin-6G LET were used. They are based on a Hamamatsu S2744 PIN diode with a sensitive area of 21.2 mm × 11.2 mm and a charge-sensitive preamplifier to measure the particle flux, the absorbed dose in silicon D_Si_, and the corresponding dose rate. Liulin detectors have been widely used in aviation by several research groups for many years^28^.

### Flight LH2574/2575

After the decision to resort to the Falkland Islands for the exchange of crew and equipment, two corresponding flights were planned for January and March 2021 (ex HAM 2021–01–31, return to MUC on 2021–02–04 and ex HAM 2021–03–30, return to MUC on 2021–04–03). However, the SARS-CoV-2 pandemic imposed extraordinary constraints on the realization of this flight mission. All crew members involved, i.e., from Lufthansa, AWI, and DLR, had to undergo comprehensive infection prevention measures. A strict quarantine in a controlled hotel exclusively organized for this purpose starting a fortnight before the flight, and three negative SARS-CoV-2 sensitive PCR tests during that time were required in order to board the flight to the Falkland Islands.

The assigned flight numbers were LH2574 for the flight from Hamburg to Mount Pleasant (East Falkland) and LH2575 for the return flight to Munich. The essential measurements of the radiation exposure in the geographical region of the SAA were taken on flight LH2574 ex HAM on 2021–03–30. Since the exchanged AWI crew was quite small and no equipment with weight worth mentioning was transported, the payload was extremely low on this flight. Therefore, the aircraft could already climb to the Flight Level (FL) of 43,000 ft., also referred to as FL430, shortly after crossing the equator and before reaching the region of interest and remained at this altitude for the rest of the flight. Furthermore, the flight mission Atlantic Kiss was performed with the most modern aircraft of Lufthansa’s A350 fleet at that time (registration D-AIXQ). The touchdown of LH2574 took place at Mount Pleasant Airport on 2021–03–31 at 1120 UTC after a flight time of 15 h 46 min, which also set a new record in the history of Lufthansa. Upon arrival on East Falkland, the crews of Lufthansa and DLR had to undergo a strict room quarantine in a selected hotel in Stanley during the two-day layover again while the AWI crew was transferred to the research vessel Polarstern that was anchored in Stanley Harbor.

### Space weather conditions

**Figure 2:**
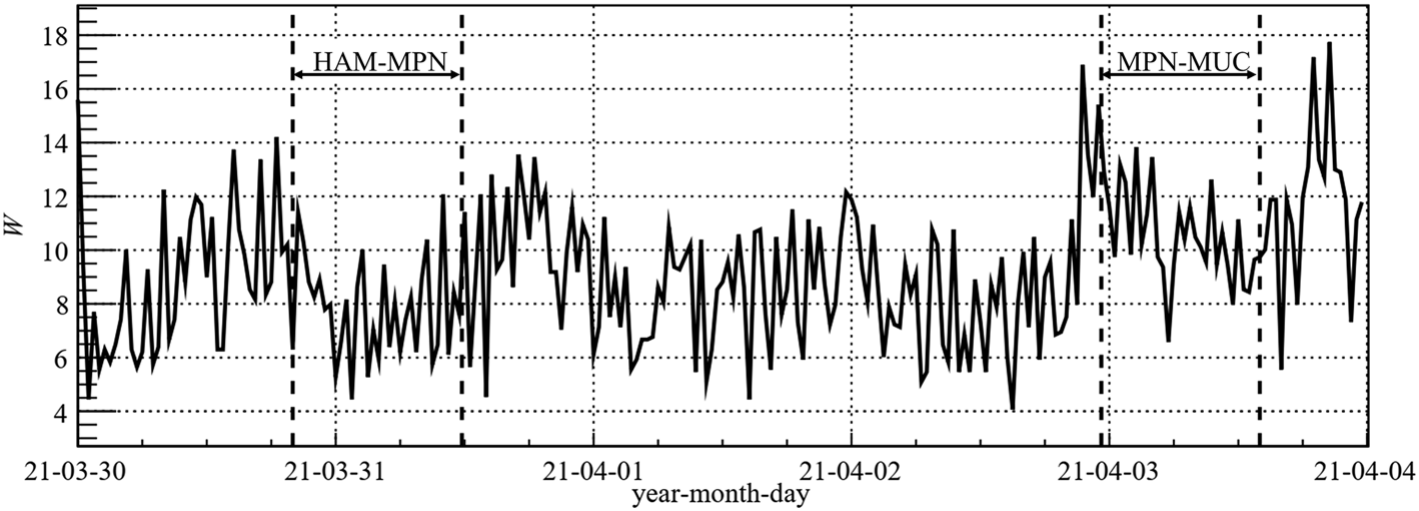
Variation of the W-parameter based on 30-min averages of the Oulu Neutron Monitor count rates. Departure and arrival times of the flights are indicated by the dashed vertical lines.

The flight mission Atlantic Kiss through the geographical region of the SAA was originally planned for the solar minimum between cycle 24 and 25 in 2020. During solar minima, the exposures from GCR at flight altitudes are at maximum which provides optimal measuring, i.e., counting statistics within the solar cycle. Furthermore, space weather conditions are typically particularly stable in terms of magnetospheric disturbances and the probability of solar particle events is extremely small during this transitional period. The influence of the solar activity can be expressed by the W-parameter, a model parameter using the GCR-model by Matthiä et al.^29^. Figure 2 shows the variation of the W-parameter, derived from the 30-min averaged count rates of the Oulu Neutron Monitor (NM), for both flights HAM-MPN and MPN-MUC. The NM count rates averaged over the flights were 6784 min^−1^ and 6753 min^−1^ and the derived model parameters were *W* = 7.8 and *W* = 10.7 for HAM-MPN and MPN-MUC, respectively. The corresponding variation of the derived primary particle fluence rate during the flights was within the expected statistical fluctuations for quiet space weather conditions, i.e., on the order of a few percent and below 1% for the Oulu Neutron Monitor count rates, which show variations of 10–25% between solar maximum and minimum.

Solar particle events or geomagnetic storms that might have disturbed the radiation field at aviation altitudes as well as the radiation belts were not observed during the flights. The particle flux was below 1 pfu (particle flux unit, 1 pfu = 1 particle per second per cm^2^ per steradian) according to NOAA’s Space Weather Highlights SWPC PRF 2379 of 5 April 2021. Moreover, the acquisition of reference data requires an undisturbed magnetosphere. Disturbances of the magnetosphere can be described by the Kp-index, which ranges from 0 to 9. Quiet magnetospheric conditions are assumed if this index is not greater than 3^1^. The Kp-index during the flight from HAM to MPN was ≤ 3- and it was ≤ 1 + during the flight from MPN to MUC^30^. Thus, the space weather conditions were optimal for the acquisition of reference data during the flight mission Atlantic Kiss.

## Results

**Figure 3:**
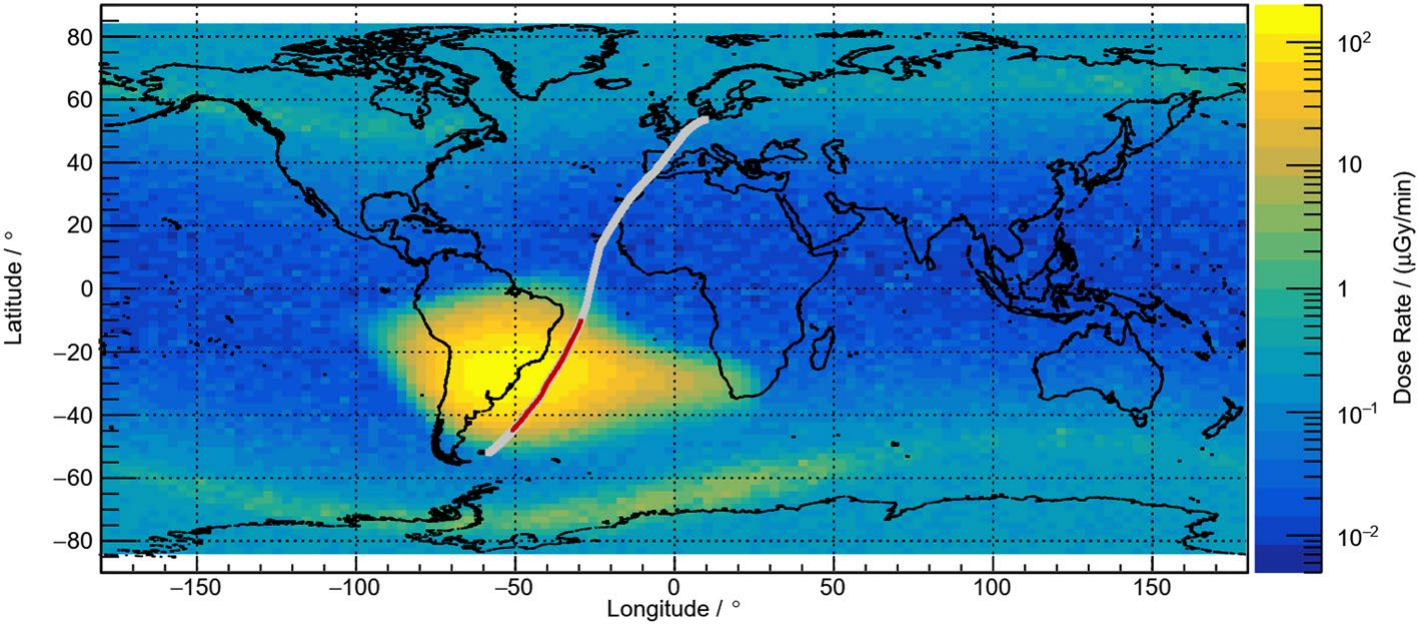
Absorbed dose rate in silicon (µGy/min) at 600 km altitude measured with the RAMIS instrument aboard the DLR Eu:CROPIS satellite in March and April 2021. The gray line represents the flight trajectory of the Atlantic Kiss mission from HAM to MPN. The red section of the flight route is considered as region of interest.

**Figure 4:**
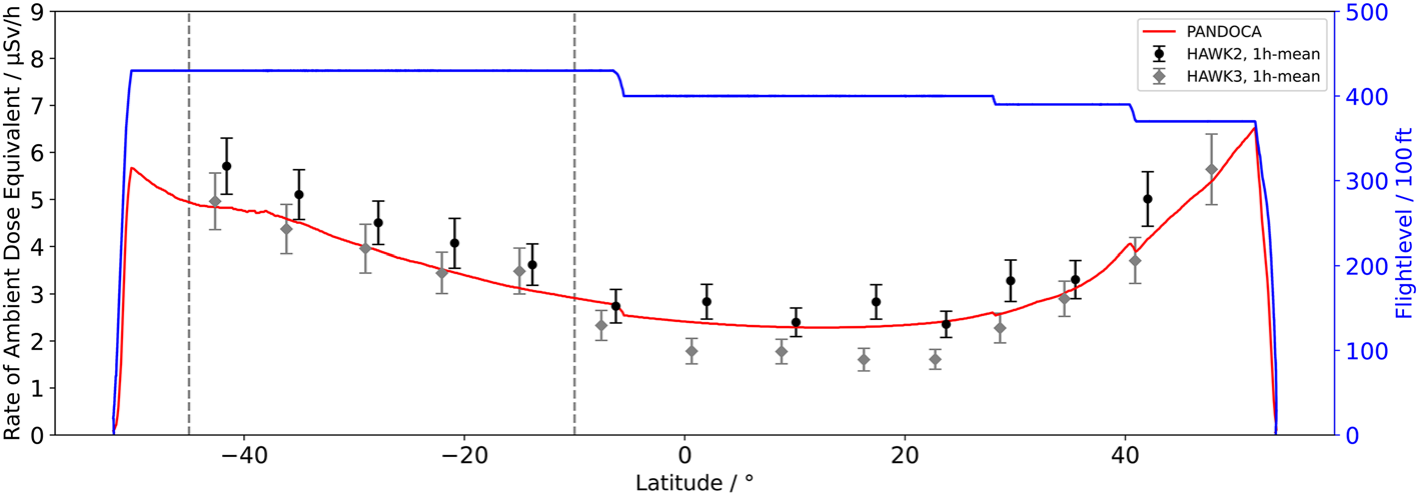
HAWK 2 and HAWK 3 data of the ambient dose equivalent rate in comparison with PANDOCA model calculations (red line). The flight profile is shown as well (blue line).

**Figure 5:**
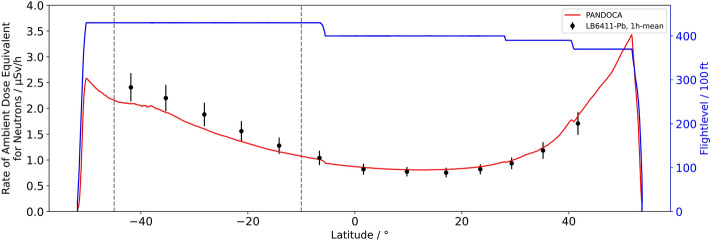
The rate of ambient dose equivalent for neutrons dH_N_/dt in dependence on the geographic latitude for the PANDOCA model and the measuring values acquired using the Neutron Probe LB6411-Pb (1 h mean). The flight profile is shown as well (blue line).

**Figure 6:**
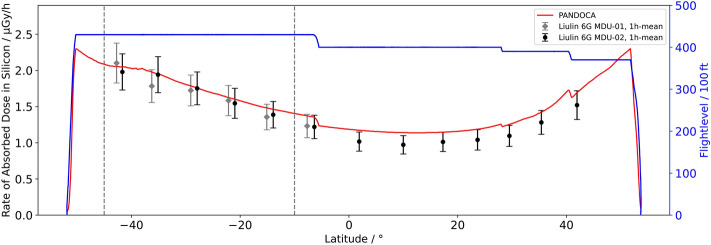
The rate of the absorbed dose in silicon in dependence on the geographic latitude for the PANDOCA model and the measuring values acquired using the Liulin MDU-1 and MDU-2 (1 h mean). The flight profile is shown as well (blue line).

The relevant measuring data were acquired during flight LH2574 from HAM to MPN since the geographical region of the SAA was entirely crossed at FL430. However, the core area of this region changes in size and position depending on the altitude, e.g., the orbit of a satellite used for recording reference data. Consequently, the region of interest for the flight trajectory of LH2574 had to be defined as the range of latitudes between − 10° and − 45°, i.e., 10°S and 45°S, where the dose rate measured aboard the Eu:CROPIS satellite was considerably higher than anywhere else (cf. Fig. 3). The flight duration in this region was 5:04 h. Most of the devices used measure temporally resolved dose rates. In order to make the measured values of these dose rates easier to compare, in particular within the region of interest, the corresponding data are correlated to the flight data with high resolution, which allows to plot the data with regard to latitude rather than time. Additionally, 1-h means of dose rates are calculated for each instrument to reduce statistical uncertainties. The latitude-dependent dose rates for the neutron probe LB6411-Pb, the Liulin-6G instruments, and the two HAWKs are shown in Figs. 4, 5 and 6. In addition, the corresponding model calculations with PANDOCA are compared with the measurements. The flight levels as well as the boundaries of the region of interest (dashed lines), as defined above, are also plotted for reference. The given uncertainties for the measurements include both the statistical uncertainties of the mean values and the assumed systematic uncertainties for the individual instruments. The generally accepted level of uncertainty when comparing model calculations with measuring data in the domain of aviation dosimetry is 30%^31^.

### HAWK

The systematic uncertainties for both HAWKs are assumed to be on the order of 5%^32^. The measuring data of the ambient dose equivalent rate of the HAWK version 2 are slightly higher than the PANDOCA model calculations for all latitudes and altitudes of LH2574. This behavior reflects a systematic deviation and is also in good agreement with the results of the CONCORD flight campaign (COmparisoN of COsmic Radiation Detectors^28^). The data acquired by HAWK version 3 are lower than the model calculations in the equatorial region, but show good agreement otherwise (Fig. 4). Neither HAWK shows any evidence for an additional significant contribution to the radiation field at aviation altitudes from the SAA.

### Neutron probe LB6411-Pb

The data of the Neutron Probe LB6411-Pb and the PANDOCA calculations of the ambient dose rate by neutrons dH_N_/dt show overall a good agreement (Fig. 5). The systematic uncertainties of the instrument are estimated to be about 10%^33^. From high latitudes to low latitudes the calculated dose rate shifts from being slightly over the measurement to being slightly under it. This seems to be a combined effect of latitude, i.e., geomagnetic shielding, as well as altitude and is no special feature of the data in the SAA region. If there were a considerable contribution from the SAA in the region of interest, an increase in dose rates above the GCR background toward the center of the SAA at around -30° would be expected followed by a decrease further south in contrast to the observed monotonically increasing underestimation of model dose rates. Especially at the southern end of the region of interest no such decline of dose rate is measured. Therefore, it is highly unlikely that the observed deviation between modeled and measured dose rates is related to an additional neutron contribution to the radiation field at FL430 due to the SAA.

### Liulin-6G

The Liulin-6G measuring data and the PANDOCA model calculations are overall in good agreement (Fig. 6). The systematic uncertainties of the two Liulin-6G Mobile Dosimetry Units (MDU) used are estimated to be about 10%^32^. Liulin MDU-1 was intentionally not switched on before crossing the equator on LH2574 in order to have a fully charged spare instrument available for data acquisition in the region of interest. The measuring values match the calculations with no discernible excess in the SAA region which indicates that the instrument did not measure unknown contributions to the radiation field due to charged particles.

### Integral dose

The total dose values for the flights from HAM to MPN (LH2574) and from MPN to MUC (LH2575) are listed in Table 1 that also includes the integral doses for two additional semiconductor dosimeters of type M-42 developed at DLR^34^. The neutron measurements were supported by Bubble Detectors (BDs)^35,36^, the results of which are listed in Table 1 as well. On flight LH2575 from MPN to MUC only BDs with low sensitivity were used resulting in a dose value with high statistical uncertainty. Since Liulin MDU-1 was only switched on after crossing the equator, the total dose in silicon is not available for this flight. The particular dose values of the dose quantities used are different for LH2574 and LH2575 due to the different flight profiles and duration.

**Table 1:** Comparison of total doses and model calculations.

	HAM–MPN LH2574	MPN–MUC LH2575
Ambient dose equivalent H*(10) [µSv]		
HAWK2	61 ± 4	54 ± 3
HAWK3	51 ± 3	44 ± 3
PANDOCA	54.0	46.3
Neutron component H_N_ of H*(10) [µSv]		
LB6411-Pb	23 ± 2	18 ± 2
Bubble detectors	22 ± 5	47 ± 20
PANDOCA	22.5	18.7
Absorbed dose in silicon D_Si_ [µGy]		
Liulin 6G MDU-1	−	20 ± 2
Liulin 6G MDU-2	22 ± 2	19 ± 2
M42-004	23 ± 2	20 ± 2
M42-006	22 ± 2	19 ± 2
PANDOCA	23.9	21.0

## Discussion

The results of the measuring data acquired during the Atlantic Kiss mission do not give any indication of an impact of the inner Van Allen radiation belt on the radiation exposure in the geographical region of the SAA at a flight altitude of 43,000 ft., i.e., 13 km or below. Actually, this outcome is not surprising, since no sufficiently efficient mechanism for the transport of radiation generated by protons with usual energies from the inner radiation belt down to aviation altitudes is known. The trajectory of the trapped charged particles is a gyro-motion in a spiral along the magnetic field lines with decreasing pitch toward lower altitudes. The particles reach a mirror point at the lowest altitude and bounce back toward higher altitudes. The minimum altitude above ground depends on various variables, e.g., the velocity of the particle, its initial pitch angle, and the geographic location, which is the reason why the inner Van Allen radiation belt reaches down to lower altitudes compared to other geographical regions due to the tilt and shift of the dipole axis of the geomagnetic field. Since even in the SAA region, the mirror points of the protons are at altitudes above 100 km, the particles do not reach the atmosphere. While numerous events during which electrons were deviated from their trajectory in the outer radiation belt and precipitated into the atmosphere are documented, e.g., Clilverd et al.^37^, it is unclear if similar events occur during which a large number of high-energy protons from the inner radiation belt enter the atmosphere. However, even if high-energy protons entered the atmosphere, their range would be quite limited. An analysis of proton energy spectra, observed in the SAA by the Energetic Particle Telescope (EPT) aboard the ESA (European Space Agency) satellite PROBA-V (Project for On-Board Autonomy -Vegetation), has shown a decrease in proton fluxes by about two orders of magnitude in the energy range between 100 and 250 MeV at different positions within the SAA at an altitude of 820 km^38^. A vertically incident proton would require an energy of some 550 MeV to reach FL430 due to the atmospheric shielding independent of the geomagnetic shielding. Furthermore, the AP8 radiation belt model does not predict any proton flux with energies above 500 MeV in the radiation belts at an altitude of 300 km or below and no proton flux at energies above 100 MeV at an altitude of 150 km or below (cf. AP8-min as implemented in SPENVIS (SPace ENVironment Information System), www.spenvis.oma.be). Additionally, it is not expected that a significant fraction of the charged particles that are trapped in the inner radiation belt enters the atmosphere at all, especially at low zenith angles. For this reason, the Van Allen belts have not been included in the PANDOCA model as a source that might affect the radiation exposure of aircrew and passengers. A theoretical radiation transport mechanism might be conceivable due to the creation and atmospheric transport of a secondary neutron field. Although this cannot be ruled out a priori, it would require a comparatively high primary proton flux. Therefore, the experimental investigation of the neutron component is crucial to exclude this radiation transport mechanism.

The measuring data of the Atlantic Kiss mission confirm the PANDOCA model calculations within the generally accepted level of uncertainty very well. Several radiation measurements onboard aircraft were already taken in the South Atlantic region, e.g., by DLR for the verification of the PANDOCA model^21^ and by Federico et al.^9^ as fixed point and route missions during the previous transition from solar cycle 23–24 under similar space weather conditions. Neither flight mission gave any indication of locally increased dose rates in the geographical region of the SAA. The Atlantic Kiss mission, however, offered the unique opportunity to cross the entire region of interest with an A350 at an altitude of 13 km in a single flight.

The results are based on measurements acquired during quiet space weather conditions in transition from solar cycle 24–25 and corresponding measurements should be repeated during times of strong solar activity, i.e., over a period of disturbed magnetosphere for additional confirmation.

## Conclusions

The mission Atlantic Kiss was planned to investigate any potential effect of the inner Van Allen radiation belt on the radiation exposure at the altitudes of civil aviation in the geographical region of the SAA and could be finally performed on a unique flight from HAM to MPN in 2021 during the transition between solar cycle 24 and 25 despite the worldwide SARS-CoV-2 pandemic crisis. The operational conditions for this mission were optimal since the entire geographical region of interest could be crossed at an altitude of 13 km during quiet space weather. Furthermore, the calculations using the radiation model PANDOCA have been confirmed with the measuring data of the investigated dose quantities for this altitude too, i.e., the SAA does not cause an additional measurable contribution to the expected radiation exposure due to the GCR component at the flight altitudes of civil aviation under stable space weather conditions. The urban legend of increased radiation exposure at flight altitudes in the geographical region of the SAA seems to be based on the assumption of a linear or similar relationship between the increase in the radiation exposure in LEOs inside and outside the SAA and the corresponding effect on the atmosphere beneath the inner Van Allen radiation belt. Moreover, this widely spread misconception is seemingly supported by increased levels of ionizing radiation even at ground level. However, the strong absorption of particles from the radiation belt due to the atmospheric shielding is ignored in this concept and the measured increase in radiation on the ground has been proven to be caused by terrestrial radioactivity, e.g., ^232^Th^13,14^. Furthermore, the measuring data taken on the flight LH2574 at an altitude of 13 km are consistent with the atmospheric radiation transport of protons observed in the SAA aboard the PROBA-V satellite using the radiation transport model PANDOCA and confirm previous radiation measurements onboard aircraft in the South Atlantic region by Federico et al. and their comparison with different radiation transport models^9^.

In summary, the results of the mission Atlantic Kiss contribute to debunking the urban legend of generally increased levels of ionizing radiation at flight altitudes in the geographical region of the SAA that caused unnecessary concern among crew members and passengers.

## Data Availability

The datasets generated during and/or analyzed during the current study are available from the corresponding author on reasonable request.
